# Modeled Respiratory Tract Deposition of Aerosolized Oil Diluents Used in Δ^9^-THC-Based Electronic Cigarette Liquid Products

**DOI:** 10.3389/fpubh.2021.744166

**Published:** 2021-11-04

**Authors:** Anand Ranpara, Aleksandr B. Stefaniak, Kenneth Williams, Elizabeth Fernandez, Ryan F. LeBouf

**Affiliations:** Respiratory Health Division, National Institute for Occupational Safety and Health, Morgantown, WV, United States

**Keywords:** e-cigarette, EVALI, vitamin E acetate, particle size distributions, lung deposition, secondhand exposure estimates

## Abstract

Electronic cigarette, or vaping, products (EVP) heat liquids (“e-liquids”) that contain substances (licit or illicit) and deliver aerosolized particles into the lungs. Commercially available oils such as Vitamin-E-acetate (VEA), Vitamin E oil, coconut, and medium chain triglycerides (MCT) were often the constituents of e-liquids associated with an e-cigarette, or vaping, product use-associated lung injury (EVALI). The objective of this study was to evaluate the mass-based physical characteristics of the aerosolized e-liquids prepared using these oil diluents. These characteristics were particle size distributions for modeling regional respiratory deposition and puff-based total aerosol mass for estimating the number of particles delivered to the respiratory tract. Four types of e-liquids were prepared by adding terpenes to oil diluents individually: VEA, Vitamin E oil, coconut oil, and MCT. A smoking machine was used to aerosolize each e-liquid at a predetermined puff topography (volume of 55 ml for 3 s with 30-s intervals between puffs). A cascade impactor was used to collect the size-segregated aerosol for calculating the mass median aerodynamic diameter (MMAD) and geometric standard deviation (GSD). The respiratory deposition of EVP aerosols on inhalation was estimated using the Multiple-Path Particle Dosimetry model. From these results, the exhaled fraction of EVP aerosols was calculated as a surrogate of secondhand exposure potential. The MMAD of VEA (0.61 μm) was statistically different compared to MCT (0.38 μm) and coconut oil (0.47 μm) but not to Vitamin E oil (0.58 μm); *p* < 0.05. Wider aerosol size distribution was observed for VEA (GSD 2.35) and MCT (GSD 2.08) compared with coconut oil (GSD 1.53) and Vitamin E oil (GSD 1.55). Irrespective of the statistical differences between MMADs, dosimetry modeling resulted in the similar regional and lobular deposition of particles for all e-liquids in the respiratory tract. The highest (~0.08 or more) fractional deposition was predicted in the pulmonary region, which is consistent as the site of injury among EVALI cases. Secondhand exposure calculations indicated that a substantial amount of EVP aerosols could be exhaled, which has potential implications for bystanders. The number of EVALI cases has declined with the removal of VEA; however, further research is required to investigate the commonly available commercial ingredients used in e-liquid preparations.

## Introduction

Electronic cigarette, or vaping, products (EVP) work by aerosolizing a liquid that is inhaled into the lungs by the user. The liquid used in an EVP, also known as e-liquid, can contain humectants, nicotine, flavorings, and other types of chemicals. EVP can be modified to aerosolize e-liquids that contain various forms of cannabis plant extracts, oil diluents, and other substances and additives. One of these extracts, delta-9-tetrahydrocannabinol (Δ^9^-THC), used in some e-liquids, contains mind-altering psychoactive properties that give a “high” (1). Along with reports that most EVALI patients (85%) were 18 years or older, ever use of Δ^9^-THC among youth (8.9%), and use of Δ^9^-THC among EVP users (30.6%), are indicative of potential health risks in the United States (2–5). Perrine et al. (6) stated that among college students, 75% of EVP users consumed various products of cannabis extracts in e-liquids. He et al. first reported a case of acute respiratory failure in a person who inhaled aerosolized Δ^9^-THC in 2017 (7). Subsequently, in 2019, the United States experienced an epidemic of acute lung injury termed as “e-cigarette or vaping use-associated lung injury” (EVALI) among persons who reportedly inhaled aerosolized Δ^9^-THC or nicotine e-liquids (8). As of January 14, 2020, among 2,668 hospitalized EVALI cases or deaths, about half were younger than 24 years, and 82% reported using an EVP to inhale Δ^9^-THC (9–11).

Δ^9^-THC extracts are hydrophobic, highly viscous, and semi-solid, and require thinning by dilution to be used in e-liquids aerosolized by EVP. Oils such as Vitamin-E acetate (VEA), Vitamin E oil, medium chain triglycerides (MCT), and coconut oil are used to dilute Δ^9^-THC extracts to create e-liquids. Heating these diluents to aerosolize Δ^9^-THC oils produces harmful chemicals, such as acetone, duroquinone, durohydroquinone, short chain esters, short chain alkanes, and ethenone (12–16).

Blount et al. (13, 14) measured several possible toxic substances as exposure markers in bronchoalveolar lavage fluid (BALF) samples from EVALI patients and an unaffected comparison group. These included Δ^9^-THC and e-liquid constituents such as VEA, MCT, coconut oil (identified as a common MCT), and terpenes such as limonene. VEA, coconut oil, and limonene were quantified in 94, 2, and 3% of EVALI-patient BALF samples, respectively, but were not detected in BALF samples from a comparison group. Among these oil diluents, tasteless, and odorless VEA is likely preferred by manufacturers because its viscosity profile makes it difficult to differentiate between pure Δ^9^-THC extracts and diluted products (14, 17).

A joint investigation conducted by the Centers for Disease Control and Prevention (9), the U.S. Food and Drug Administration, and the state health authorities identified VEA as strongly linked to the clinical presentation among EVALI cases and it also led to pulmonary damage *in vivo* using a mouse model (18). Although EVALI appeared to resolve by stopping the use of VEA, other diluents could potentially play a role or have other toxic effects (9, 10, 19). While studies have shown the formation of potentially toxic gases when these diluents were heated, information is lacking on the physical and chemical properties of inhaled aerosol particles after aerosolizing diluent oils (20).

EVP aerosol is a two-phase mixture of gases and particles (21–23). Sosnowski and Kramek-Romanowska (24) highlighted the need to understand the size distribution of inhaled EVP aerosols as an influential factor for estimating their regional deposition in the respiratory tract. Other studies have mentioned sites of regional lung depositions for inhaled, and fractions for exhaled, micron-sized particles (25, 26). Measurement of the size distribution of the EVP aerosol can be challenging because aerosolized liquid droplets change their native size, depending on various conditions such as evaporation and hygroscopic growth. Evaporation of liquid droplets in the EVP aerosol during sampling results in an under-estimation of particle size, while hygroscopic growth results in an over-estimation of particle size (23). These deviations in size distribution, in turn, result in errant predictions of regional deposition in the respiratory tract. The native physical and chemical properties of particles should be maintained as intact as possible during measurement to determine the accurate size distribution of the emitted EVP aerosol (27, 28). Oldham et al. (27) predicted gas and particle phases as a function of the mass of collected aerosols without dilution, and therefore unadulterated mass-based aerosol size distribution is considered as an important parameter to determine their lung deposition (29, 30).

Recently, one study has assessed particle size distribution of aerosolized VEA from a commercially available EVP using a combination of a differential mobility spectrometer and an electrical low-pressure impactor. The authors noticed a substantial decrease in particle size for three out of four tested vape-pens, a type of commercially available EVP, because of air dilution caused by high puffing flow rates (31). In the current study, mass-based particle size distribution was directly measured with as little dilution as possible for several common oil diluents used as a constituent of EVP e-liquids for inhalation of Δ^9^-THC. We then estimated the location and mass concentration of deposited inhaled EVP aerosols in the respiratory tract. In addition, we estimated the exhaled fraction as secondhand exposure fraction, which can potentially affect the health of the bystanders and workers at certain occupational settings, such as vape shops and smoking centers.

## Materials and Methods

### Diluents and Simulated e-Liquid Preparation

VEA was purchased from Sigma–Aldrich (St. Louis, MO, United States), and the Vitamin E oil (42,900 IU, 100% pure & natural, Chandler, AZ, United Sates), MCT (100% organic unflavored, Garden of Life LLC, FL, United Sates), and coconut oil (Organic unflavored, Carrington Farms, Closter, NJ, United Sates) were purchased from Amazon (Seattle, WA, United Sates). To more closely mimic herein prepared simulated e-liquids with commonly used e-liquids, these oils were thinned with ethanol (200 proof, ACS/USP grade, CAS# 64-17-5, Pharmaco-Aaper, Brookfield, CT, United Sates) at 0.6% w/w and with terpenes: *d*-limonene (ACS grade, CAS# 5969-27-5, Sigma–Aldrich) at 0.2% w/w and α-pinene (ACS grade, CAS# 80-56-8, Sigma–Aldrich) at 0.2% w/w. We chose to simulate e-liquid formulation because ethanol is a solvent commonly used to extract and solubilize Δ^9^-THC oils, and terpenes are used to make Δ^9^-THC miscible in e-liquids (14, 17). Simulated e-liquids were prepared gravimetrically using a Mettler Toledo XS 205 dual-range microbalance capable of measuring to 0.01 mg (Mettler-Toledo LLC, Columbus, OH, United States) and homogenized for 1 h using a ThermoScientific rotator, Model 4152110 (Dubuque, IA, United States). The density of the diluent oils was measured in grams per cubic centimeter (g/cm^3^): Vitamin E oil (1.21 g/cm^3^), VEA (0.96 g/cm^3^), coconut oil (0.94 g/cm^3^), and MCT (0.91 g/cm^3^).

### Experimental Setup

The U.S. National Institute of Drug Abuse has developed a Standardized Research E-Cigarette (SREC) and considered NJOY (NJOY Inc., Scottsdale, AZ, United States) as a reference EVP (32). Studies reported the prevalent use of “Dank Vapes” among EVALI cases, which are Δ^9^-THC-containing pre-filled cartridges that operate below one ohm of resistance (33, 34). We used NJOY top tanks in our study because they are refillable and compatible with the sub-ohm resistance of “Dank Vapes” devices although they are a different EVP brand. NJOY top tanks, Model # UVTB02, can be filled with 1.6 ml of e-liquid. An automated e-cigarette aerosol generator (ECAG; e~Aerosols LLC, Central Valley, NY, United States) was programmed to aerosolize each simulated e-liquid. The ECAG works on positive pressure to aerosolize the simulated e-liquids by heating the coil at 3.7 volts (set) of electric current at a determined puff topography. Puff topography was calibrated daily to 55 ml puff volume within 3 s (1 puff) with a 30-s puff delay (35), using a soap-bubble flow meter (Borgwalt KC GmbH, Hamburg, Germany) as a primary volumetric flow calibration device. Three puffs were directly sampled without dilution into a MiniMOUDI (MSP Corporation, Shoreview, MN, United States), a type of low-flow cascade impactor, to preserve the native physical and chemical properties of the aerosol intact (27, 28). The MiniMOUDI was used to size fractionate e-cigarette aerosol (size range: 0.056–10 μm) at a sampling flow rate of 2 liters per minute (LPM). The mass of aerosols deposited on each impactor stage at cut off particle diameter (Dp: 0.056–10 μm)] was measured on a 37-mm aluminum filter using a Mettler Toledo XS 205 dual range microbalance with a mass resolution of 0.01 mg (Mettler-Toledo LLC, Columbus, OH, United States).

A new NJOY tank was used to fill 1.3 ± 0.5 ml for each laboratory prepared e-liquid, which was puffed for 3 min before conducting the trials. Five trials were conducted for each of the e-liquids. The second set of five trials was conducted with a single VEA e-liquid preparation to assess reproducibility across each day of testing. There was no significant difference (*p*-value = 0.19) between the average mass median aerodynamic diameter (MMAD) of aerosolized VEA across days (0.71 μm on day 1 and 0.61 μm on day 2), which indicated that the size distribution based on mass for VEA was reproducible.

**Figure 1:**
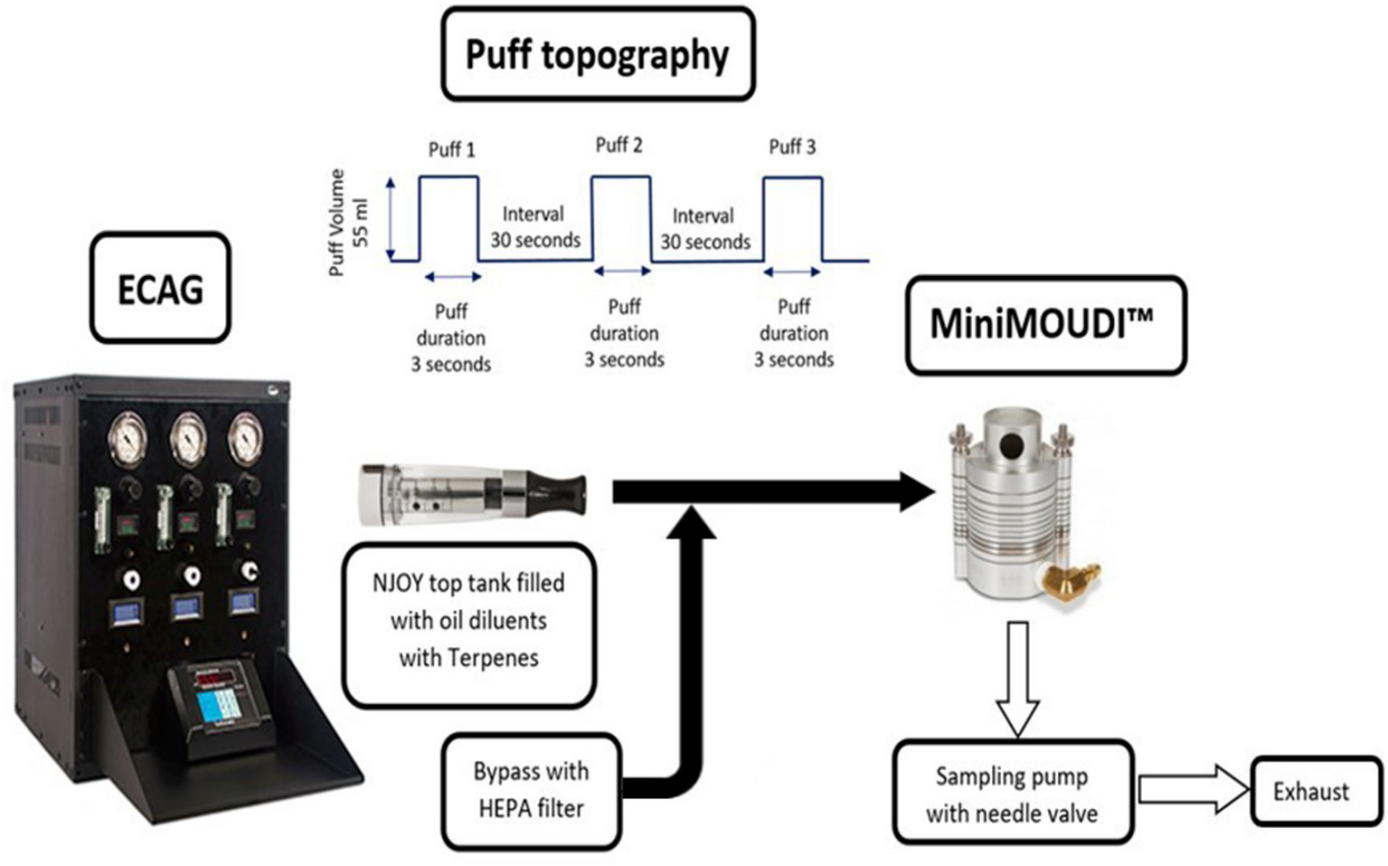
Schematics of the experimental setup.

Figure 1 shows a schematic of the experimental setup. The ECAG provided power to the device and forced air with an established puff topography through the tank into the MiniMOUDI. When the ECAG was operating, 1.1 LPM of EVP aerosol was sent directly to the MiniMOUDI along with 0.9 LPM of high-efficiency particulate air (HEPA: Whatman Schleicher & Schuell; Stockbridge, GA, United States)-filtered bypass air. During the puff delay, the impactor sampled 2.0 LPM from the bypass air that did not result in any mass loading on the aluminum filters for any size of aerosols. To avoid aerosol losses, the mouthpiece of the tank was connected to the inlet of the MiniMOUDI using a small piece of flexible, black conductive silicone tubing with an inside diameter of 0.5 cm. A bypass HEPA-air filter was attached to allow uninterrupted flow to the impactor and to alleviate pressure drops and volume flow differences between the aerosol supply and the sampler requirements.

### Statistical Analyses

Data were log transformed and analyzed using JMP 13.0 (SAS Institute Inc., Cary, NC, United States). To determine particle size distributions, the MMAD and GSD were calculated by including each cutoff size of the MiniMOUDI using a probit model for five trials on each e-liquid. To consider the significant differences (*p* < 0.05) between the particle size distribution of e-liquids, the average MMAD (*n* = 5) between e-liquids was compared using a one-way ANOVA and Tukey's HSD multiple comparisons. Deposited mass (in mg) of EVP aerosols for each size bin (Dp) was calculated by measuring aluminum substrate before (pre) and after (post) sampling. The total deposited mass of e-liquid aerosol was calculated by adding the mass collected at each particle cut-off diameter (μm) from Dp_i_ = 0.056–10 for every trial (*n* = 5). Puff mass (mg/puff) was calculated for e-liquids according to Equation 1. Consideration of total mass collection per puff-based particle size distribution was intended to allow for the comparison between studies that researched mass-based particle size distribution.


(1) $$\begin{array}{l} Puff\;mass\;\left(\frac{mg}{puff}\right)for\;every\;trial\;(n=5)=\\ \left[\frac{\sum_{Dpi=0.056}^{10}\left(Post\;mass\left(mg\right)-Pre\;mass\left(mg\right)\right)}{\#of\;puffs}\right] \end{array}$$

### Lung Deposition Modeling

Based on the MMAD and GSD, the fraction of inhaled particles that could deposit in different sites of the human respiratory tract was predicted using the Multiple-Path Particle Dosimetry (MPPD), version 3.04 (ARA, Albuquerque, NM).

Estimates for regional deposition as the fraction of inhaled EVP aerosols were considered according to the Yeh–Schum model. The Yeh–Schum single-path model considers the whole human lung as a symmetric tree; therefore, respective regional depositions are average values for three regions: the head, trachea—bronchial (TB), and pulmonary regions (36). Regional deposition in the head includes mouth, nose, larynx, and pharynx to the trachea (generation 0). The TB region is from the trachea (generation 0) to the bronchioles (generation 16). The pulmonary region is from the terminal bronchioles onward to the alveoli. Deposition estimates are average values for each generation. Total respiratory tract deposition was calculated by summing three regional depositions. Based on the predicted deposition fractions, we modeled the conceptual estimation of EVP aerosol mass concentration (mg/puff) deposited in the respiratory tract as a product of deposition fraction and puff mass yield (Equation 1). For example, the mass of EVP aerosol deposited in the head region was modeled by multiplying the regional deposition fraction in the head with puff mass (mg/puff) yield for each e-liquid. Based on the total respiratory tract deposition upon inhalation by the EVP user, we could also estimate the fraction of EVP aerosol that is potentially exhaled out. The exhaled particles fraction was estimated using Equation 2. Both the estimated exhaled EVP aerosol fraction and the modeled mass concentrations could serve as indicators of potential secondhand exposure.


(2) $$\begin{array}{l} \;Secondhand\;exposure\;fraction=\\ 1-Total\;respiratory\;deposition\;fraction \end{array}$$

Unlike regional deposition, Yeh–Schum lobular deposition pattern characterized the segmental bronchi within each lobe as a single symmetric path to report the mass deposited in each of the five lobes of the human lungs: right upper (RU), right middle (RM), right lower (RL), left upper (LU), and left lower (LL) (36). The total lobar deposition includes deposition in the TB and pulmonary regions of each lung lobe but not the initial airways as they do not belong to any lobe. Default parameters for Yeh–Schum model were as follows: forced residual capacity = 3,300 ml, upper respiratory tract volume = 50 ml, breaths per minute (bpm) = 12, and tidal volume = 625 ml.

## Results

**Figure 2:**
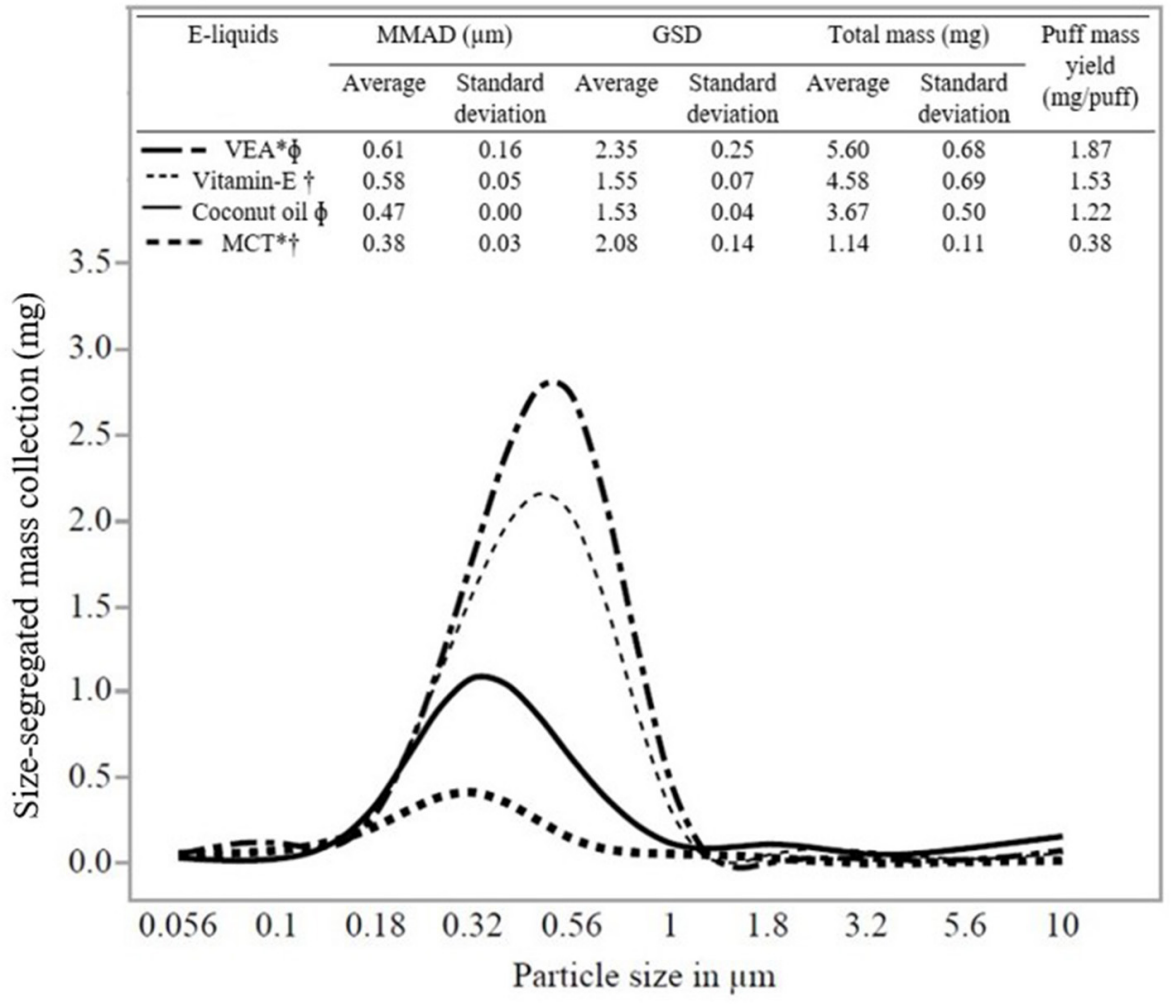
Size distribution (MMAD and GSD), puff mass yield, and statistical comparisons of e-liquids. Note that the total (non-size-segregated) mass presented at the top is different from the size-segregated mass collection presented in the y-axis. E-liquids connected by the same symbols are significantly different. ^*^VEA and MCT are significantly different. ^Φ^VEA and coconut oil are significantly different. ^†^Vitamin E oil and MCT are significantly different.

Figure 2 shows a representative particle size distribution (in the *x*-axis) and size-segregated mass (mg) deposition (in the *y*-axis) for all the e-liquids evaluated in the study. The average MMAD and standard deviation for five trials evaluating e-liquids were as follows: Vitamin E containing e-liquids (VEA: 0.61 ± 0.16 μm) and Vitamin E oil (0.58 ± 0.05 μm) and without Vitamin E containing e-liquids (coconut oil: 0.47 ± 0.00 μm) and MCT (0.38 ± 0.03 μm). One-way ANOVA (*p* = 0.0012) and Tukey's test resulted in a statistically significant difference (at *p* < 0.05) between the MMADs for VEA and MCT, VEA, and coconut oil, and Vitamin E oil and MCT (Supplementary Table S2). However, we detected no significant statistical difference between the MMADs of VEA and Vitamin E oil (*p* = 0.24). Additionally, there was a wider aerosol size distribution emitted by e-liquids for VEA (GSD 2.35) and MCT (GSD 2.08) compared with coconut oil (GSD 1.53) and Vitamin E oil (GSD 1.55). Results of MMAD and GSD values for individual trials for all the e-liquids are presented in Supplementary Table S1.

Data (average, standard deviation for *n* = 5) in Figure 2 shows the total mass and puff mass yield (mg/puff) of EVP aerosol emitted by all the e-liquids. We found that both these attributes correlated with the MMADs of the e-liquids. MMAD of VEA was the largest (0.61 μm) and resulted in the highest averaged total mass collected and puff mass yield for VEA (5.60 mg) compared with other e-liquids. Additionally, VEA and Vitamin E oil (VEA at 5.60 mg and Vitamin E oil at 4.58 mg) resulted in the comparable total mass collection and puff mass yield (VEA: 1.87 mg/puff and Vitamin E oil: 1.53 mg/puff). Because of the potential hygroscopic nature of VEA particles, larger-sized EVP aerosols have more condensable material available for particle mass-growth, which corresponds well with the finding of the higher mass collection with larger MMAD (23, 24, 37, 38). MCT and coconut oil aerosolized into smaller particles and because they have lower liquid densities (relative to VEA and vitamin E oil), they resulted in comparably less mass collection. The results of mass collection for the individual trials for all the e-liquids are presented in Supplementary Table S1.

Table 1 presents the predicted regional and total respiratory deposition (inhaled), as well as secondhand exposure (exhaled) fraction of EVP aerosols for the various e-liquids. Dosimetry modeling predicted that, out of the total respiratory deposition, higher fractions of particles were estimated to deposit in deeper lung regions (pulmonary and TB) compared with the head region. Among these e-liquids, VEA had the highest total respiratory tract deposition (0.26). Out of the total respiratory tract depositions, the majority (~0.14–0.17) of the aerosolized e-liquids were estimated to deposit in the TB (~0.06) and pulmonary (~0.08–0.11) regions.

**Table 1:** Predicted^*^ deposition fraction and modeled doze deposition for e-liquids.

**E-liquids**	Predicted* fraction					**Modeled mass (mg/puff)**				
	**Regional deposition**			**Total deposition**	**Secondhand exposure**	**Regional deposition**			**Total deposition**	**Secondhand exposure**
	**Head**	**TB**	**Pulmonary**			**Head**	**TB**	**Pulmonary**		
VEA	0.09	0.06	0.11	0.26	0.74	0.17	0.11	0.21	0.49	1.38
Vitamin E oil	0.05	0.06	0.09	0.20	0.80	0.08	0.09	0.14	0.31	1.22
Coconut oil	0.04	0.06	0.08	0.18	0.82	0.05	0.07	0.10	0.22	1.00
MCT	0.05	0.06	0.10	0.21	0.79	0.02	0.02	0.04	0.08	0.30

* *As per MPPD, version 3.04 (ARA, Albuquerque, NM)*.

Modeled mass (mg/puff) = predicted fraction ^*^ puff-mass yield (mg/puff).

Based on the predicted deposition fractions (Table 1) and calculated puff mass yield (Figure 2), modeled EVP mass concentrations (~mg/puff) for regional, total, and secondhand exposure are presented in Table 1. For e-liquids, puff mass yields (mg/puff) were as follows: VEA (1.87), Vitamin E oil (1.53), coconut oil (1.22), and MCT (0.38), and therefore were predicted to deposit more mass per puff in the respiratory tract (total and regional) of the EVP user as well as exhalation for secondhand exposure conditions. Out of the total inhaled EVP mass concentrations (~0.08–0.49 mg/puff), considerable amounts were deposited in the deeper (pulmonary and TB) lung regions, ~0.08–0.33 mg/puff, compared with the head region (~0.02–0.16 mg/puff) for all the e-liquids. For e-liquids studied in this work, the total mass inhaled per puff estimated to deposit in pulmonary regions (mg/puff) were: VEA (0.21/0.49), Vitamin E oil (0.14/0.31), coconut oil (0.10/0.22), and MCT (0.04/0.08) compared with the head and TB regions.

Indication of considerable physical deposition of herein studied EVP aerosols into the TB and pulmonary regions (predicted fraction: ~0.14–0.17 and modeled mass concentration: ~0.08–0.33 mg/puff) could explain respiratory illnesses, including BALF investigations associated with EVALI (13, 14, 17). Dosimetry analysis calculations indicated that MCT (MMAD: 0.38 μm) had a high proportion of pulmonary region deposition (~0.10) out of the total respiratory tract deposition (~0.21) because of its smaller size. However, compared to other e-liquids, estimates for MCT (puff mass yield: 0.38 mg/puff) translated to less mass concentration inhaled (~0.08 mg/puff) and deposited in pulmonary regions (~0.04 mg/puff). One of the other possibilities for these smaller-sized particles was reported to be exhaled with greater chances than larger-sized particles (25, 26). Consideration of exhalation of the smaller-sized particles is addressed in this study by presenting estimates of secondhand exposure fractions. Like MCT, similar observations were noticed for coconut and vitamin E oils regarding higher predicted pulmonary deposition fraction (~0.08 or more) and modeled mass concentrations (~ 0.10–0.14 mg/puff) and considerable secondhand exposure conditions (~0.80 or more).

Note that the predicted total respiratory tract deposition estimates and the secondhand exposure condition are inversely related, that is, the lower the total respiratory tract deposition fraction, the higher the secondhand exposure fraction. For example, with coconut oil only a small fraction of the particles estimated to account for total respiratory tract deposition (fraction: ~0.18 and mass concentration: ~0.22 mg/puff) so more particles were estimated to be exhaled out (fraction: ~0.82 and mass concentration: ~1.00 mg/puff). For all the e-liquids, the predicted total respiratory tract deposition fraction was 0.21 ± 0.04 and the estimated secondhand exposure fraction was ≥0.74. MCT resulted in the smallest MMAD and the least total mass collection, but modeling projected a considerable amount (~0.10/0.21) of emitted MCT particles that would deposit in the pulmonary region. Because of its smaller size, deposition of particles emitted by aerosolizing MCT is predicted deep in the pulmonary region and exhaled out as well, which explains estimates for the regional deposition, lower total respiratory tract deposition (~0.21), and total mass concentration (~0.08 mg/puff). It is noteworthy that we did not detect a significant difference between MMAD of vitamin E oil (0.58 μm) and VEA (0.61 μm), and like VEA (~0.11), a considerable amount of total inhaled vitamin E oil aerosols (~0.20) was also predicted to deposit in the pulmonary region (~0.09). Additionally, a large fraction (~0.80) of aerosolized vitamin E oil particles could potentially account for secondhand exposure conditions. Exhaled EVP aerosols fraction and mass per puff can be a potential indicator of secondhand exposure conditions for bystanders including those in occupational settings.

**Figure 3:**
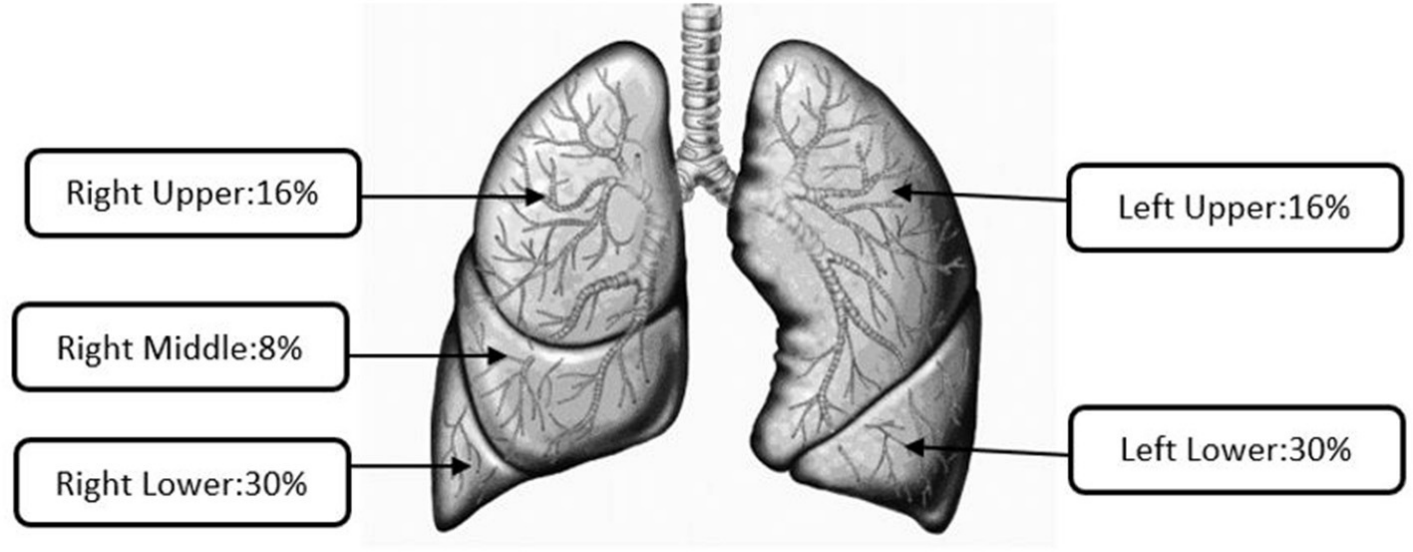
Average lobular deposition for all e-liquids.

Figure 3 presents the average lobular aerosol mass depositions of all the studied e-liquids. However, higher percentages of the aerosol mass were found to be deposited in right-sided lung lobes (RU, RM, and RL = 54%) compared with the left-sided lung lobes (LU and LL = 46%). Manigrasso et al. have presented right lung lobes as the sites where effects of the EVP aerosol occur more likely than left lung lobes (39). The highest percentages of lobular deposition of emitted aerosols from all the oils were predicted in the lower lobes (right: 30 ± 0.2% and left: 30 ± 0.2%) compared to other lobes of the lungs (RU 16 ± 0.2%; RM 8 ± 0.1%; and LU 16 ± 0.2%). For particle sizes in the range from 0.2 to 1 μm, higher deposition fractions in lower lobes compared to the upper lobes were also documented, as observed in our results and prior studies (36, 40–43).

## Discussion

In the CDC update on February 2020, 68 confirmed deaths were reported out of a total of 2,807 hospitalized EVALI cases (9). Studies have quantified some toxic chemicals present in BALF at the time of clinical examination from EVALI cases and confirmed that VEA was strongly linked to EVALI (9, 14, 17). However, the physiological mechanism by which aerosolized diluent oils found in BALF of EVALI patients actually injured lungs remains unclear. Not only does the pathophysiology of these chemically-induced damages remain unclear, but adequate research on the physical characteristics of the EVP aerosols, necessary to understand regional lung depositions, has, until now, been lacking.

Mikheev et al. (31) evaluated the particle size distribution and chemical composition of aerosolized VEA using four commercially available vape pens. This group measured particle size distribution by sampling eight puffs of aerosolized VEA with a 60-s puff interval, which required high flow rates (20–40 LPM). The two puff flow rates considered in the study of Mikheev et al. were 20 ml/s and 40 ml/s for 5 s with a 60-s puff delay. Although particle size distribution was also dependent on the types of commercial EVP devices and their heating capability, the authors noticed a strong influence of puffing flow rate on the size of aerosolized VEA particles that resulted in particle sizes smaller than 50 nm at the higher puffing flow rate (40 ml/s) (31). In contrast to the study of Mikheev et al., we sampled three puffs of EVP aerosols at a puff flow rate of 18.33 ml/s with a 30-s puff delay with no dilution flow. Our study focused on directly measuring mass-based particle size, to the extent feasible without deviation, from their native size aerosolized from simulated e-liquids. The intention was to mimic the composition of oil diluent constituents consistent with products associated with EVALI cases but without the presence of Δ^9^-THC for safety reasons. Rather than using commercially available e-liquids, we prepared e-liquids in the laboratory that contained each diluent oil, to evaluate the specific influence of that diluent oil on MMAD and lung deposition. This study showed no statistical difference (*p* = 0.24) between the MMAD values of VEA (0.61 μm) and Vitamin E oil (0.58 μm), which translated into similar estimates regarding respiratory deposition behavior. Compared to MCT and coconut oil, the aerosolization of Vitamin E-containing e-liquids (VEA and Vitamin E oil) was observed to report larger MMADs and a greater total mass of EVP aerosol.

Variations in EVP aerosol generation and characterization methods have led to a lack of reproducibility. Therefore, the ability to compare various studies or to integrate information is difficult (44–46). The puff-based mass collection presented in this study was an attempt to compare the results between existing studies within the given experimental parameters. Using the same type of cascade impactor, (37) found size distributions of two commercial e-cigs comparable to the size distributions measured by Ingebrethsen et al. (38) using spectral extinction with a slightly variable puff topography. As presented in Figure 2, we observed a similar trend between MMAD and mass collected for all the studied e-liquids. For VEA with MMAD of 0.61 μm, puff mass yield resulted in 1.87 mg/puff, which is comparable with the study of Alderman et al. results for one commercially available e-cig with MMAD of 0.63 μm that yielded puff mass of 2.16 mg/puff. Apart from different experimental parameters and sampling methods, differences were noticed in puff-mass measurements between these two studies for the same EVP devices. It was also hypothesized that the possibility of growth for larger-sized particles resulted in more mass collection. Similar to the study of Alderman et al., we observed more mass collection [Puff mass yield: VEA (1.87 mg/puff), vitamin E oil (1.53 mg/puff), coconut oil (1.22 mg/puff), and MCT (0.38 mg/puff)] for larger MMAD [VEA (0.61 μm), vitamin E oil (0.58 μm), coconut oil (0.47 μm), and MCT (0.38 μm)] of diluent oils, respectively. Our laboratory-prepared e-liquids included common diluent oils but not polypropylene glycol (PG), vegetable glycerin (VG), or nicotine. Particle size distribution of EVP aerosol depends on e-liquid compositions. This being one of the first studies to address the mass-based direct measurement of the particle size distribution for diluent oils, it is important to present the information in a way that could be compared with further research considering different experimental parameters. Moreover, by considering the accurate measurement of native particle size as close as possible, we provided conceptual estimates for deposition fraction and modeled per puff mass–dose deposition.

Out of the total respiratory tract deposition for all the diluent oils, the highest fraction (~0.08–0.11) and mass per puff (mg/puff: 0.04–0.21) were predicted in the pulmonary region where gas exchange occurs. Lewis et al. (29) noted that a maximum probability of particle deposition in the pulmonary region was for particles with MMAD <3 μm and GSD <3. For smaller-sized particles with MMAD <2 μm, Raabe et al. (47) concluded that the highest fraction of total deposited particles is reported in the pulmonary region. Dosimetry results presented in this study for VEA were consistent with these inhalation toxicological evaluations. We observed that VEA resulted in the highest total mass collected (5.60 mg) *via* cascade impactors and the highest total respiratory tract deposition (~0.26) *via* dosimetry analysis, compared with all the e-liquids included in the study. Literature showed the higher lobular depositions of submicron size particles in lower lobes than upper lobes of the lungs (36, 40–43), which is the same as our study results. Using number concentrations, Manigrasso et al. estimated size-segregated aerosols emitted from commercially available EVP as a function of the airway generation number in the lung lobes. They concluded that, for both the TB and pulmonary regions, twice as many particles were deposited in RU compared to the LU and ~0.20 more particles deposited in RL compared to LL. Cumulatively, right lung and lobar bronchi were documented as sites where PG–VG-based e-liquid aerosols may likely affect more than the left lung, which is consistent with our study results.

It is possible that pulmonary deposition of constituents of aerosolized e-liquids alters airway homeostasis, changes surfactant integrity, and provokes oxidative or inflammatory damages or contributes to the formation of lipid-laden macrophages (14, 48–50). These histological findings might be consistent with BALF investigations and clinical presentations, such as chemical pneumonitis, among EVALI cases (14, 51). Two research studies conducted in mice presented similar lung pathologies to EVALI patients following the inhalation of aerosolized VEA (18, 52).

Jiang et al. (15) reported that the aerosolization of MCT, Vitamin E oil, and VEA yielded the cytotoxic products short chain esters, duroquinone, and durohydroquinone, respectively, one or more of which might explain cellular damages among the reported EVALI cases (15). In the current study, a greater fraction of aerosolized MCT (~0.10), VEA (~0.11), and Vitamin E oil (~0.09) were predicted to deposit in the pulmonary region of the lung. If one or more of the cytotoxic products reported by Jiang et al. contribute to EVALI, our data support this exposure pathway based on these similar respiratory particle deposition patterns. Regarding MCT, a fraction (~0.21) of aerosolized particle sizes <1 μm were estimated to be deposited in the respiratory tract, which could be a reason why coconut oil was observed in BALF samples for one of the EVALI cases (reference).

Herein discussed assessments aligning with the available literature indicate that particles smaller than 1 μm can deposit in the pulmonary region of the lung and are also exhaled from the lung with greater probability than particles larger than 1 μm (25, 26). These exhaled particles could serve as a source of potential secondhand exposure to nearby people (53, 54). Secondhand exposures are not only important in maintaining indoor air quality for bystanders at domestic settings where vulnerable populations are in proximity to EVP users but also at certain occupational settings such as vape shops (20, 53, 54). In that regard, the greater fraction (~0.74 or more) of the aerosols emitted from all the e-liquids studied were estimated to be a source of potential secondhand exposure. This high predicted fraction of exhaled particles could also explain why analyses found coconut oil (compared to VEA) in the BALF sample of only one EVALI case (the lowest total respiratory tract deposition at ~0.18 and highest second-hand exposure fraction at ~0.82) (13, 14, 17). Direct measurement of exhaled aerosols should be conducted to determine a true particle size distribution that could contribute to secondhand exposure because the primary aerosol inhaled by an EVP user likely differs from the secondhand aerosol exhaled by a user.

### Study Limitations

The results of this laboratory study are limited to one fixed puff topography from the CORESTA method: 3 s puff, 30 s interval, and 55 ml puff volume. However, without standardized experimental protocols, parameters included in any study could be a source of limitation, which also causes a lack of reproducibility. At times, contrasting observations were noticed between studies that addressed the effects of the EVP settings, such as voltage, power, and coil resistance, that influence heating of e-liquids, on the particle size of the EVP aerosol that is generated and lung depositions (55–59). Unlike Floyd et al. and Lechasseur et al., Mulder et al. purported that the compositions of e-liquids, such as the various proportions of PG–VG, significantly impact particle size distributions, and not the voltage and coil resistance. Studies also showed that commercially available e-liquids do not have their ingredients and their proportion of ingredients fully documented, which limits their influence on conclusions drawn (60–65). Therefore, in this study, we prepared e-liquids in the laboratory that contained each diluent oil with terpenes to validate their influence on particle emission after heating at 3.6 V with a particular puff topography. Li et al. (66) evaluated the effects of heating PG–VG-based e-liquids at different puff volumes, puff duration, and interval on the particle size of aerosols and mass deposition in the respiratory tract. One of their puff profiles was exactly what we used in this study, but further investigations are necessary to evaluate the effects of puff profile on the particle size distribution and lung deposition using e-liquids containing diluent oils. We addressed the particle size distribution of diluent oils in e-liquids intended to mimic products used by EVALI cases and revealed that more experiments regarding different puff topographies are needed in the future.

For any study of EVP aerosols, the determination of the particle size distribution in its native state is complex because of the dynamics involved in generating and measuring a mist from a variety of EVP devices (23). Protano et al. (45) demonstrated that there were significant variations in puff-to-puff EVP aerosol generation within a single device with all other parameters held constant. Considering impaction and spectral transmissions, various studies using different EVP models and brands, reported particle sizes that ranged from 0.1 to 0.9 μm and utilizing sampling methods without the dilution of the emitted aerosol (24, 37, 38, 67). Using a low-flow impactor, MMADs observed in our studies for all e-liquids ranged from 0.38 to 0.61 μm, which was comparable with the study results of Alderman et al. (MMAD range: 0.43–0.63 μm) for similar puff topography (with 3 s puff, 30 s interval, and 50 ml of puff volume). MMAD results (0.38–0.61 μm) presented in this study using NJOY top tank sub-ohm EVP devices were comparable with previous reports (37, 38).

The EVP construction material such as ceramic vs. non-ceramic coil used to heat e-liquid could be a source of variability that influences the size distribution of aerosol e-liquids. VEA and the other oils tested are more typically aerosolized using a ceramic cell EVP device that usually functions at a higher temperature than a sub-ohm resistance device. One limitation of our study is that we evaluated a sub-ohm resistance NJOY top tank EVP device, not a ceramic cell device, which could modify the size distribution generated for the prearranged puff topography.

Finally, the estimated respiratory deposition fractions using MPPD were not modified for hygroscopic growth and evaporation according to the human lung environment. Dosimetry analysis did not consider various factors such as aerosol temperature, hygroscopicity, relative humidity, and gas–vapor interchange, all of which can impact modeled lung deposition from the oral cavity throughout the respiratory tract (68–70). Our approach was to accurately measure the native size distribution of particles emitted after aerosolizing diluent oils and to predict respiratory deposition fraction and mass–dose per puff. Future studies should incorporate, to the extent feasible, these factors that influence deposition into models (where available) to provide more accurate dose estimates. Within the presented experimental parameters, our results bolster ongoing EVALI investigations as well as provide valuable data on the physical deposition of particles in the deep regions of the respiratory tract, which, when coupled with toxicological investigations associated with diluent oils in e-liquids, provided insights into the disease.

## Conclusions

Diluent oils such as VEA, Vitamin E oil, coconut oil, and MCT are mixed with Δ9-THC extracts, so the thinned down products can be used as a constituent of e-liquids. Although various toxicological investigations and histopathological studies have reported evidence of lung damage from inhalation of these diluent oils in aerosolized e-liquids, particle size distribution, which is necessary to understand regional depositions in the respiratory tract, has not been addressed. This study focused on determining and comparing particle size distributions of aerosol emitted from simulated e-liquids that contained VEA, Vitamin E oil, coconut oil, or MCT with terpenes. Based on MMADs, particle size distribution for VEA (0.61 μm) was significantly different than coconut oil (0.47 μm) and MCT (0.38 μm) but not Vitamin E oil (0.58 μm).

Dosimetry analysis predicted that ~60% of total respiratory depositions of particles were in the pulmonary (~42%) and TB (~20%) regions for VEA. Irrespective of statistical difference in their size distribution, aerosolized particles were predominantly (~69% or more) deposited in lower lobes (Right: ~30% and Left: ~30%) of the lungs. These observed particle deposition patterns were consistent with previous inhalation toxicological studies and with characterization of BALF of EVALI cases, which support the pulmonary region of the lung as the site of injury. The study results presented herein help to explain existing clinical presentations and pathological findings by providing particle size distribution of diluent oils and their respiratory depositions. Additionally, EVP aerosol sizes less than 1 μm, which have high probability of being inhaled then exhaled, could pose secondhand exposure risk to persons in proximity to EVP users in occupational and non-occupational settings. While elimination of VEA in e-liquid products seemed to mitigate the EVALI outbreak, further research is required to investigate the usage of other commonly available oil diluents in Δ9-THC-based e-liquid preparations, which could also be potentially harmful for users and bystanders.

## Data Availability Statement

The original contributions presented in the study are included in the article/Supplementary Material, further inquiries can be directed to the corresponding author/s.

## Author Contributions

All authors listed have made a substantial, direct and intellectual contribution to the work, and approved it for publication.

## Funding

This work was supported by NIOSH intramural research funds.

## Author Disclaimer

The findings and conclusions in this report are those of the authors and do not necessarily represent the official position of the National Institute for Occupational Safety and Health, Centers for Disease Control and Prevention. Mention of any company or product does not constitute an endorsement by the U.S. Government, the National Institute for Occupational Safety and Health, or the Centers for Disease Control and Prevention.

## Conflict of Interest

The authors declare that the research was conducted in the absence of any commercial or financial relationships that could be construed as a potential conflict of interest.

## Publisher's Note

All claims expressed in this article are solely those of the authors and do not necessarily represent those of their affiliated organizations, or those of the publisher, the editors and the reviewers. Any product that may be evaluated in this article, or claim that may be made by its manufacturer, is not guaranteed or endorsed by the publisher.

## References

[1] Breitbarth AK Morgan J Jones AL. E-cigarettes—an unintended illicit drug delivery system. Drug Alcohol Depend. (2018) 192:98–111. 10.1016/j.drugalcdep.2018.07.031 30245461

[2] Cullen KA Gentzke AS Sawdey MD Chang JT Anic GM Wang TW. E-cigarette use among youth in the United States. JAMA. (2019) 322:2095–103. 10.1001/jama.2019.18387 31688912 PMC6865299

[3] Singh T Arrazola RA Corey CG Husten CG Neff LJ Homa DM. Tobacco use among middle and high school students—United States, 2011–2015. MMWR Morb Mortal Wkly Rep. (2016) 65:361–7. 10.15585/mmwr.mm6514a1 27077789

[4] Trivers KF Phillips E Gentzke AS Tynan MA Neff LJ. Prevalence of cannabis use in electronic cigarettes among US youth. JAMA Pediatr. (2018) 17211:1097–9. 10.1001/jamapediatrics.2018.1920 30242366 PMC6248134

[5] Trivers KF Gentzke AS Phillips E Tynan M Marynak KL Schauer GL. Substances used in electronic vapor products among adults in the United States, 2017. Addict Behav Rep. (2019) 10:100222. 10.1016/j.abrep.2019.100222 31828201 PMC6888746

[6] Perrine C Pickens C Boehmer T King B Jones C DeSisto C. Characteristics of a multistate outbreak of lung injury associated with e-cigarette use, or vaping—United States. Morb Mortal Wkly Rep. (2019) 68:860–4. 10.15585/mmwr.mm6839e1 31581168 PMC6776378

[7] He T Oks M Esposito M Steinberg H Makaryus M. “Tree-in-Bloom”: severe acute lung injury induced by vaping cannabis oil. Ann Am Thorac Soc. (2017) 14:468–70. 10.1513/AnnalsATS.201612-974LE 28248584

[8] Schier JG Meiman JG Layden J Mikosz CA VanFrank B King BA. Severe pulmonary disease associated with electronic-cigarette-product use—interim guidance. MMWR Morb Mortal Wkly Rep. (2019) 68:787–90. 10.15585/mmwr.mm6836e2 31513561 PMC6755818

[9] Centers for Disease Control and Prevention. Outbreak of Lung Injury Associated With the Use of e-Cigarette, or Vaping, Products. (2020). Cincinnati, OH: U.S. Department of Health and Human Services, Centers for Disease Control and Prevention. Available online at: https://www.cdc.gov/tobacco/basic_information/e-cigarettes/severe-lung-disease.html (accessed March 2020).

[10] Krishnasamy VP Hallowell BD Ko JY Board A Hartnett KP Salvatore PP. Update: characteristics of a nationwide outbreak of e-cigarette, or vaping, product use-associated lung injury—United States. Morb Mortal Wkly Rep. (2020) 69:90–4. 10.15585/mmwr.mm6903e2 31971931 PMC7367698

[11] Moritz ED Zapata LB Lekiachvili A Glidden E Annor FB Werner AK. Update: Characteristics of patients in a national outbreak of e-cigarette, or vaping, product use-associated lung injuries—United States, October 2019. Morb Mortal Wkly Rep. (2019) 68:985–9. 10.15585/mmwr.mm6843e1 31671085 PMC6822806

[12] Attfield KR Chen W Cummings KJ Jacob P O'Shea DF Wagner J. Potential of ethenone (ketene) to contribute to electronic cigarette, or vaping, product use–associated lung injury. Am J Respir Crit Care Med. (2020) 202:1187–9. 10.1164/rccm.202003-0654LE 32551843

[13] Blount BC Karwowski MP Morel-Espinosa M Rees J Sosnoff C Cowan E. Evaluation of bronchoalveolar lavage fluid from patients in an outbreak of e-cigarette, or vaping, product use-associated lung injury−10 states, August–October 2019. MMWR Morb Mortal Wkly Rep. (2019) 68:1040–1. 10.15585/mmwr.mm6845e2 31725707 PMC6855513

[14] Blount BC Karwowski MP Shields PG Morel-Espinosa M Valentin-Blasini L Gardner M. Vitamin E acetate in bronchoalveolar-lavage fluid associated with EVALI. N Engl J Med. (2020) 382:697–705. 10.1056/NEJMoa1916433 31860793 PMC7032996

[15] Jiang H Ahmed CMS Martin TJ Canchola A Oswald IWH Garcia JA. Chemical and toxicological characterization of vaping emission products from commonly used vape juice diluents. Chem Res Toxicol. (2020) 33:2157–63. 10.1021/acs.chemrestox.0c00174 32618192

[16] Troutt WD DiDonato MD. Carbonyl compounds produced by vaporizing cannabis oil thinning agents. J Altern Complement Med. (2017) 23:879–84. 10.1089/acm.2016.0337 28355118

[17] Duffy B Li L Lu S Durocher L Dittmar M Delaney-Baldwin E. Analysis of cannabinoid-containing fluids in illicit vaping cartridges recovered from pulmonary injury patients: identification of vitamin E acetate as a major diluent. Toxics. (2020) 8:8. 10.3390/toxics8010008 31991538 PMC7151740

[18] Bhat TA Kalathil SG Bogner PN Blount BC Goniewicz ML Thanavala YM. An animal model of inhaled Vitamin E acetate and EVALI-like lung injury. Engl J Med. (2020) 382:1175–7. 10.1056/NEJMc2000231 32101656 PMC7299285

[19] Taylor J Wiens T Peterson J Saravia S Lunda M Hanson K. Characteristics of e-cigarette, or vaping, products used by patients with associated lung injury and products seized by law enforcement—Minnesota, 2018 and 2019. Morb Mortal Wkly Rep. (2019) 68:1096–100. 10.15585/mmwr.mm6847e1 31774740 PMC6881051

[20] Stefaniak AB LeBouf RF Ranpara A Leonard SS. Toxicology of flavoring- and cannabis-containing e-liquids used in electronic delivery systems. Pharmacol Ther. (2021) 224:107838. 10.1016/j.pharmthera.2021.107838 33746051 PMC8251682

[21] Pankow JF. Calculating compound dependent gas-droplet distributions in aerosols of propylene glycol and glycerol from electronic cigarettes. J Aerosol Sci. (2017) 107:9–13. 10.1016/j.jaerosci.2017.02.003 31213727 PMC6581467

[22] Pankow JF Kim K Luo W McWhirter KJ. Gas/particle partitioning constants of nicotine, selected toxicants, and flavor chemicals in solutions of 50/50 propylene glycol/glycerol as used in electronic cigarettes. Chem Res Toxicol. (2018) 31:985–90. 10.1021/acs.chemrestox.8b00178 30113826 PMC6513566

[23] Sosnowski TR Jablczynska K Odziomek M Schlage WK Kuczaj AK. Physicochemical studies of direct interactions between lung surfactant and components of electronic cigarettes liquid mixtures. Inhal Toxicol. (2018) 30:159–68. 10.1080/08958378.2018.1478916 29932004

[24] Sosnowski TR Kramek-Romanowska K. Predicted deposition of e-cigarette aerosol in the human lungs. J Aerosol Med Pulm Drug Deliv. (2016) 29:299–309. 10.1089/jamp.2015.1268 26907696

[25] Chow AHL Tong HHY Chattopadhyay P Shekunov BY. Particle engineering for pulmonary drug delivery. Pharm Res. (2007) 24:411–37. 10.1007/s11095-006-9174-3 17245651

[26] Haughney J Price D Barnes NC Virchow JC Roche N Chrystyn H. Choosing inhaler devices for people with asthma: Current knowledge and outstanding research needs. Respir Med. (2010) 104:1237–45. 10.1016/j.rmed.2010.04.012 20472415

[27] Oldham M Zhang J Rusyniak MJ Kane DB Gardner WP. Particle size distribution of selected electronic nicotine delivery system products. Food Chem Toxicol. (2018) 113:236–40. 10.1016/j.fct.2018.01.045 29408542

[28] Zhao J Zhang Y Sisler JD Shaffer J Leonard SS Morris AM. Assessment of reactive oxygen species generated by electronic cigarettes using acellular and cellular approaches. J Hazard Mater. (2018) 344:549–57. 10.1016/j.jhazmat.2017.10.057 29102637 PMC5848214

[29] Lewis TR Morrow PE McClellan RO Raabe OG Kennedy GL Schwetz BA. Establishing aerosol exposure concentrations for inhalation toxicity studies. Toxicol Appl Pharmacol. (1989) 99:377–83. 10.1016/0041-008X(89)90147-6 2749728

[30] OECD. Environment, Health, and Safety Publications. Series on Testing and Assessment. (2002). No. 39 B. Draft guidance document on acute inhalation toxicity testing. Organization for Economic Co-operation and Development. Available online at: http://www.oecd.org/chemicalsafety/testing/2765785.pdf. (accessed March 20, 2021).

[31] Mikheev VB Klupinski TP Ivanov A Lucas EA Strozier ED Fix C. Particle size distribution and chemical composition of aerosolized vitamin E acetate. Aerosol Sci Technol. (2020) 54:993–8. 10.1080/02786826.2020.1783431 33132476 PMC7595293

[32] NIDA. NIDA Drug Supply Program. (2017). Available online at: https://www.drugabuse.gov/research/research-data-measures-resources/nida-drug-supply-program/ (accessed March 20, 2021).

[33] Balmes JR. Vaping-induced acute lung injury: an epidemic that could have been prevented. Am J Respir Crit Care Med. (2019) 200:1342–4. 10.1164/rccm.201910-1903ED 31613146 PMC6884057

[34] Ghinai I Pray IW Navon L O'Laughlin K Saathoff-Huber L Hoots B. E-cigarette product use, or vaping, among persons with associated lung injury–Illinois and Wisconsin. Morb Mortal Wkly Rep. (2019) 68:865–9. 10.15585/mmwr.mm6839e2 31581166 PMC6776374

[35] Cooperation Centre for Scientific Research Relative to Tobacco. CORESTA recommended method N° 81. In: CORESTA E-Cigarette Task Force Technical Report. (2015). Available online at: https://www.coresta.org/sites/default/files/technical_documents/main/CRM_81.pdf (accessed March 2020).

[36] Yeh HC Schum GM. Models of human lung airways and their application to inhaled particle deposition. Bull Math Biol. (1980) 42:461–80. 10.1016/S0092-8240(80)80060-7 7378614

[37] Alderman SL Song C Moldoveanu SC Cole SK. Particle size distribution of E-Cigarette aerosols and the relationship to Cambridge filter pad collection efficiency. (2015) 26. 10.1515/cttr-2015-0006

[38] Ingebrethsen BJ Cole SK Alderman SL. Electronic cigarette aerosol particle size distribution measurements. Inhal Toxicol. (2012) 24:976–84. 10.3109/08958378.2012.744781 23216158

[39] Manigrasso M Buonanno G Stabile L Morawska L Avino P. Particle doses in the pulmonary lobes of electronic and conventional cigarette users. Environ. (2015) 202:24–31. 10.1016/j.envpol.2015.03.008 25796074

[40] Bennett WD Messina MS Smaldone GC. Effect of exercise on deposition and subsequent retention of inhaled particles. J Appl Physiol. (1985) 59:1046–54. 10.1152/jappl.1985.59.4.1046 4055585

[41] Chang YH Yu CP. A model of ventilation distribution in the human lung. Aerosol Sci Technol. (1999) 30:309–19. 10.1080/027868299304660 11676446

[42] Gerrity TR Garrard CS Yeates DB. Theoretic analysis of sites of aerosol deposition in the human lung. Chest. (1981) 80:898–901. 10.1378/chest.80.6.898 7307633

[43] Subramaniam RP Asgharian B Freijer JI Miller FJ Anjilvel S. Analysis of lobar differences in particle deposition in the human lung. Inhal Toxicol. (2008) 15:1–21. 10.1080/08958370304451 12476357

[44] Peace MR Haley A Mulder HA Baird TR Butler KE Friedrich AK. Evaluation of nicotine and the components of e-liquids generated from e-cigarette aerosols. J Anal Tox. (2018) 42:537–43. 10.1093/jat/bky056 30371842 PMC6203128

[45] Protano C Avino P Manigrasso M Vivaldi V Perna F Valeriani F. Environmental electronic vape exposure from four different generations of electronic cigarettes: airborne particulate matter levels. Int J Environ Res Public Health. (2018) 15:2172. 10.3390/ijerph15102172 30282910 PMC6210766

[46] Zervas E Litsiou E Konstantopoulos K Poulopoulos S Katsaounou P. Physical characterization of the aerosol of an electronic cigarette: impact of refill liquids. Inhal Toxicol. (2018) 30:218–23. 10.1080/08958378.2018.1500662 30257112

[47] Raabe OG Yeh HC Newton GJ Phalen RF Velasquez DF. Deposition of inhaled monodisperse aerosols in small *rodents*. (1975) 4:3–21. 1236165

[48] Chand HS Muthumalage T Maziak W Rahman I. Pulmonary toxicity and the pathophysiology of electronic cigarette, or vaping product, use associated lung injury. Front Pharmacol. (2020) 10:1619. 10.3389/fphar.2019.01619 31992985 PMC6971159

[49] Hickman E Herrera CA Jaspers I. Common E-cigarette flavoring chemicals impair neutrophil phagocytosis and oxidative burst. Chem Res Toxicol. (2019) 32:982–5. 10.1021/acs.chemrestox.9b00171 31117350 PMC6626492

[50] Butt YM Smith ML Tazelaar HD Vaszar LT Swanson KL Cecchini MJ. Pathology of Vaping-Associated Lung Injury. N Engl J Med. (2019) 381:1780–1. 10.1056/NEJMc1913069 31577870

[51] Mukhopadhyay S Mehrad M Dammert P Arrossi AV Sarda R Brenner DS. Lung biopsy findings in severe pulmonary illness associated with e-cigarette use (vaping): a report of eight cases. Am J Clin Pathol. (2019) 153:30–9. 10.1093/ajcp/aqz182 31621873

[52] Matsumoto S Fang X Traber MG Jones KD Langelier C Serpa PH. Dose-dependent pulmonary toxicity of aerosolized Vitamin E acetate. Am J Respir Cell Mol Biol. (2020) 63:748–57. 10.1165/rcmb.2020-0209OC 32822237 PMC7790140

[53] Flouris AD Chorti MS Poulianiti KP Jamurtas AZ Kostikas K Tzatzarakis MN. Acute impact of active and passive electronic cigarette smoking on serum cotinine and lung function. Inhal Toxicol. (2013) 25:91–101. 10.3109/08958378.2012.758197 23363041

[54] Melstrom P Koszowski B Thanner MH Hoh E King B Bunnell R. Measuring PM2.5. ultrafine particles, nicotine air and wipe samples following the use of electronic cigarettes. Nicotine Tob Res. (2017) 19:1055–61. 10.1093/ntr/ntx058 28340080

[55] Floyd EL Queimado L Wang J Regens JL Johnson DL. Electronic cigarette power affects count concentration and particle size distribution of vaping aerosol. PLoS ONE. (2018) 13:e0210147. 10.1371/journal.pone.0210147 30596800 PMC6312322

[56] Lechasseur A Altmejd S Turgeon N Buonanno G Morawska L Brunet D. Variations in coil temperature/power and e-liquid constituents change size and lung deposition of particles emitted by an electronic cigarette. Physiol Rep. (2019) 7:14093. 10.14814/phy2.14093 31140749 PMC6540444

[57] Mulder HA Patterson JL Halquist MS Kosmider L Turner JB Poklis JL. The effect of electronic cigarette user modifications and e-liquid adulteration on the particle size profile of an aerosolized product. Sci Rep. (2019) 9:10221. 10.1038/s41598-019-46387-2 31308389 PMC6629610

[58] Hua M Talbot P. Potential health effects of electronic cigarettes: a systematic review of case reports. Prev Med Rep. (2016) 4:169–78. 10.1016/j.pmedr.2016.06.002 27413679 PMC4929082

[59] Carvalho T. C. Peters J. I. Williams R. O. (2011). Review: Influence of particle size on regional lung deposition– What evidence is there? Int J Pharm. 406, 1–10. 10.1016/j.ijpharm.2010.12.040 21232585

[60] Cheng T. Chemical evaluation of electronic cigarettes. Tob Control. (2014) 23:ii11–7. 10.1136/tobaccocontrol-2013-051482 24732157 PMC3995255

[61] Crenshaw M Tefft M Buehler S Brinkman M Clark P Gordon S. Determination of nicotine, glycerol, propylene glycol and water in electronic cigarette fluids using quantitative 1H NMR. Magn Reson Chem. (2016) 54:901–4. 10.1002/mrc.4498 27495876 PMC5069187

[62] Hutzler C Paschke M Kruschinski S Henkler F Hahn J Luch A. Chemical hazards present in liquids and vapors of electronic cigarettes. Arch Toxicol. (2014) 88:1295–308. 10.1007/s00204-014-1294-7 24958024

[63] LeBouf RF Burns DA Ranpara A Attfield K Zwack L Stefaniak AB. Headspace analysis for screening of volatile organic compound profiles of electronic juice bulk material. Anal. (2018) 410:5951–60. 10.1007/s00216-018-1215-3 29974153 PMC6129974

[64] Peace M Baird T Smith N Wolf C Poklis J Poklis A. Concentration of nicotine and glycols in 27 electronic cigarette formulations. J Anal Toxicol. (2016) 40:403–7. 10.1093/jat/bkw037 27165804 PMC4946633

[65] Raymond B Collette-Merrill K Harrison R Jarvis S Rasmussen R. The nicotine content of a sample of e-cigarette liquid manufactured in the United States. J of Addict Med. (2018) 12:127–31. 10.1097/ADM.0000000000000376 29280749

[66] Li Y Cui H Wang X Si X Fan M Chen L. Effects of puffing parameters on physical properties and deposition in human respiratory tract of e-cigarette aerosols. Tob Sci & Technol. (2020) 53:10.

[67] Sundahl M Berg E Svensson M. Aerodynamic particle size distribution and dynamic properties in aerosols from electronic cigarettes. J Aerosol Sci. (2017) 103:141–50. 10.1016/j.jaerosci.2016.10.009

[68] Asgharian B Price O Rostami A Pithawalla Y. Deposition of inhaled electronic cigarette aerosol in the human oral cavity. J Aerosol Sci. (2018) 116:34–47. 10.1016/j.jaerosci.2017.11.014

[69] Asgharian B Price O Rostami A Pithawalla Y. Regional deposition of inhaled aerosol constituents from Electronic Nicotine Delivery Systems (ENDS) in the respiratory tract. J Aerosol Sci. (2018) 126:7–20. 10.1016/j.jaerosci.2018.08.006

[70] Asgharian B Hofmann W Bergmann R. Particle deposition in a multiple-path model of the human lung. Aerosol Sci Technol. (2001) 34:332–9. 10.1080/02786820119122

