# Supplementary material for: Modeled Respiratory Tract Deposition of Aerosolized Oil Diluents Used in Δ9-THC-Based Electronic Cigarette Liquid Products
Source: Front Public Health. 2021 Nov 4;9:744166. doi: 10.3389/fpubh.2021.744166 (PMC8599147; doi:10.3389/fpubh.2021.744166)
Supplement: Supplementary file 1 [file Table_1.DOCX]

**Supplementary Material**

**Table S1: Size distribution and total mass collected of e-liquids**

| E-liquids | Trial  # | MMAD (µm) | GSD | Total mass collected (mg) |
| --- | --- | --- | --- | --- |
| VEA | 1 | 0.87 | 2.17 | 5.45 |
|  | 2 | 0.52 | 2.19 | 6.28 |
|  | 3 | 0.52 | 2.25 | 6.33 |
|  | 4 | 0.67 | 2.78 | 5.07 |
|  | 5 | 0.47 | 2.38 | 4.87 |
| MCT | 1 | 0.40 | 2.00 | 1.24 |
|  | 2 | 0.35 | 2.14 | 0.97 |
|  | 3 | 0.35 | 2.20 | 1.11 |
|  | 4 | 0.40 | 2.18 | 1.22 |
|  | 5 | 0.40 | 1.88 | 1.16 |
| Coconut oil | 1 | 0.47 | 1.60 | 2.9 |
|  | 2 | 0.47 | 1.53 | 3.78 |
|  | 3 | 0.47 | 1.53 | 3.79 |
|  | 4 | 0.47 | 1.49 | 3.59 |
|  | 5 | 0.47 | 1.49 | 4.27 |
| Vitamin E oil | 1 | 0.50 | 1.64 | 3.47 |
|  | 2 | 0.55 | 1.58 | 4.35 |
|  | 3 | 0.62 | 1.44 | 5.08 |
|  | 4 | 0.62 | 1.52 | 4.96 |
|  | 5 | 0.62 | 1.56 | 5.03 |

**Table S2: Statistical significances between e-liquids**

| E-liquids | | p-Value |
| --- | --- | --- |
| VEA | MCT | 0.0012* |
| Vitamin E oil | MCT | 0.0485* |
| VEA | Coconut oil | 0.0312* |
| Vitamin E oil | Coconut oil | 0.4252 |
| Coconut oil | MCT | 0.6042 |
| VEA | Vitamin E oil | 0.6231 |

*Statistically significant differences at p<0.05
